# Replication study confirms the association between *UBAC2 *and Behçet's disease in two independent Chinese sets of patients and controls

**DOI:** 10.1186/ar3789

**Published:** 2012-03-29

**Authors:** Shengping Hou, Qinmeng Shu, Zhengxuan Jiang, Yuanyuan Chen, Fuzhen Li, Feilan Chen, Aize Kijlstra, Peizeng Yang

**Affiliations:** 1 The First Affiliated Hospital of Chongqing Medical University, Youyi Road 1, Chongqing, 400016, P. R. China; 2 Chongqing Eye Institute, Chongqing Key Laboratory of Ophthalmology, Chongqing, PR China; 3 Zhongshan Ophthalmic Center, Sun Yat-sen University, Guangzhou, PR China; 4 Eye Research Institute Maastricht, Department of Ophthalmology, University Hospital Maastricht, Maastricht, The Netherlands

## Abstract

**Introduction:**

The purpose of this study was to replicate genetic factors associated with the susceptibility to Behçet's disease (BD). We conducted a two-stage candidate genes association and functional study, involving 477 BD patients and 1,334 normal controls of Chinese Han descent.

**Methods:**

The genotyping of five candidate genes/loci, including *LOC100129342, KIAA1529, CPVL*, *UBASH3B *and *UBAC2*, were performed using TaqMan single nucleotide polymorphism (SNP) assays. Real-time PCR and luciferase reporter assay were performed to test the function of the identified promoter polymorphism. The main outcome measures were genotype frequencies and expression levels in BD patients.

**Results:**

The first-stage study results showed that *UBAC2 *(rs9513584, *Pc *= 0.018, OR = 1.4), but not *LOC100129342*, *KIAA1529*, *CPVL*, *UBASH3B *was associated with the susceptibility to BD in Chinese Han. The fine-mapping association study of *UBAC2 *identified six risk SNPs for BD in the Chinese cohort; three of them were verified in validation study (rs3825427, first-stage *P*c = 2.2 × 10^-3^, second-stage *Pc *= 9.3 × 10^-3^, combined *Pc *= 6.9 × 10^-6^; rs9517668, first-stage *P*c = 1.7 × 10^-3^, second-stage *Pc *= 0.03, combined *Pc *= 3.3 × 10^-4^; rs9517701, first-stage *P*c = 5.1 × 10^-3^, second-stage *Pc *= 9.0 × 10^-3^, combined *Pc *= 2.9 × 10^-5^; respectively). Functional analysis showed that the risk T allele of the promoter polymorphism rs3825427 had a significantly lower promoter activity than the non-risk G allele (*P *= 0.002) and a decreased expression of *UBAC2 *transcript variant 1 in peripheral blood mononuclear cells (PBMCs) and skin of normal controls carrying the risk T allele than that in individuals with the G allele (*P *= 0.045, *P *= 0.025; respectively). The mRNA expression of *UBAC2 *transcript variant 1 was significantly decreased in PBMCs and skin of BD patients as compared with controls (*P *= 0.025; *P *= 0.047, respectively). The mRNA expression of UBAC2 transcript variant 2 was significantly increased in skin of BD patients as compared with controls (*P *= 0.004).

**Conclusions:**

This study replicates a predisposition gene to BD, *UBAC2*, and suggests that *UBAC2 *may be involved in the development of BD through its transcriptional modulation.

## Introduction

Behçet's disease (BD) is generally considered as a refractory multisystem disorder characterized by recurrent oral ulceration, genital ulceration, recurrent uveitis and multiple skin lesions [1,2]. BD presents a significant geographical distribution, mainly seen in the countries along the ancient silk route [3]. It is one of the most common and severe sight-threatening uveitis entities in China [3]. Although the etiology of this disease remains unclear, it is currently thought that a genetic predisposition coupled with a triggering event seems to lead to its development. Although *HLA-B51 *has been found to be strongly associated with BD [4-7], it is estimated that *HLA-B51 *only accounts for 20% of the risk genes for this disease [8]. A whole genome linkage study has implicated several potential non-*HLA *contributory loci/genes for BD [9]. Candidate gene association studies have identified a number of susceptible genes for BD, including *IL23R *[10], *CTLA4 *[11], *eNOS *[12], *SUMO4 *[13,14], *NOD2 *[15], *IL1A *[16], manganese superoxide dismutase (*SOD*) [17] and so on. These studies tremendously enhanced our understanding of the genetic etiology of BD. However, these identified risk genes only contribute to about 30% of the predisposition for BD [18].

Fei and coworkers [19] conducted a genome-wide association study (GWAS) on 152 Turkish Behçet's disease patients using the DNA pooling approach and reported the association of BD with five novel loci/genes, including *LOC100129342*, *KIAA1529*, *CPVL*, *UBASH3B *and ubiquitin-associated domain containing 2 (*UBAC2*), among which the genetic association between *UBAC2 *and BD was lately confirmed and functional significance was tested by the same study group [20]. As far as we know, the association result needs to be confirmed by further replication studies, particularly in other ethnic groups. We, therefore, conducted a candidate association to investigate the association between the identified risk genes/loci and BD in the non-Turkish ethnic population. Our result revealed that *UBAC2 *was associated with BD in Han Chinese. Fine mapping and validation studies further confirmed *UBAC2 *as susceptibility gene for Behçet's disease. Further functional study showed that the risk T allele of rs3825427 in *UBAC2 *was associated with the decreased promoter activity and its mRNA expression. These genetic associations and functional studies suggest that *UBAC2 *is a risk gene for Behçet's disease.

## Materials and methods

### Ethics statement

Written informed consent was obtained from each participant, and this study was approved by the Clinical Research Ethics Committee of the First Affiliated Hospital of Chongqing Medical University and Zhongshan Ophthalmic Center, Sun Yat-sen University (Permit Number: 2009-201004).

### Subjects

One hundred forty-seven BD patients and 951 normal controls enrolled in the first-stage were recruited from April 2005 to March 2008 in Southern China at Zhongshan Ophthalmic Center, Sun Yat-sen University. The replication cohort is comprised of 330 Han Chinese BD patients and 383 Han Chinese normal controls, which were recruited from April 2008 to March 2010 in Western China at the First Affiliated Hospital of Chongqing Medical University (Table 1). The diagnosis of BD was based on the criteria of the International Study Group for BD [2]. Twelve patients with typical nongranulomatous uveitis, multiform skin lesions or genital ulcers in association with arthritis or other clinical features, were diagnosed as having BD according to the criteria of the Behçet's Disease Research Committee of Japan and were also included in this study (Table 2) [21]. The control populations consisted of unrelated healthy individuals from the same geographical regions as the BD patients and were age, sex and ethnically matched with the patients.

**Table 1:** Summary characteristic of Behçet's patients and normal control subjects in this study

	Case			Control		
		
Analysis	Samples size	Mean age (S.D)	Male/Female	Samples size	Mean age (S.D)	Male/Female
Stage I	147	32.90 ± 7.50	129/18	951	39.77 ± 9.99	712/239
Stage II	330	33.39 ± 7.98	268/62	383	34.61 ± 7.85	301/82
Combined	477	33.07 ± 7.64	397/80	1334	37.07 ± 9.11	1,013/321

SD, standard deviation

**Table 2:** The clinical characteristics of Behcet patients

Characteristics	BD Patients	
	Total (*n *= 477)	%
Male	362	75.9
Female	115	24.1
Uveitis	477	100
Oral ulcer	465	97.5
Genital ulcer	167	35.0
Hypopyon	111	23.3
Skin lesions	270	56.6
Positive pathergy test	167	35.0
Arthritis	135	28.3

### SNPs selection

We chose the enrolled 25 SNPs based on the following principle: we focused on the *UBAC2 *gene and ran Haploview software (Daly Lab at the Broad Institute, Cambridge, MA, USA) using Chinese Han Beijing data to screen out tagSNPs including rs3825427, rs1927726, rs9517668, rs9554581, rs4636771, rs7332161, rs9517701, rs7325747, rs912129, rs4772190 and also included the reported BD associated SNP rs9513584 [19]. In order to enhance the accuracy rating of genotyping, we also included 16 SNPs with high heterozygosity (allele frequency > 0.1).

The three SNPs, including rs3825427, rs9517668 and rs9517701, were enrolled in the replication study because of their smaller Bonferroni corrected *P-*value. The call rate of SNP rs9517699 did not meet the quality standard in the replication study (call rate < 0.80), so SNP rs9517699 was excluded in the replication analysis (Additional file 1).

### SNP genotyping

Genomic DNA was extracted from the peripheral blood of patients and controls using the QIAamp DNA Blood Mini Kit (QIAGEN Inc., Hilden, Germany) according to the manufacturer's instructions. The Genotyping was determined by TaqMan^® ^SNP Genotyping Assays (Applied Biosystems, Foster City, CA, USA) on the Applied Biosystems 7500 Real-Time PCR System according to the manufacturer's instructions. All SNPs tested in this study had a genotyping success rate > 98% and accuracy > 99% as judged by random re-sequencing of 20% of samples in all subjects.

### Real-time quantitative PCR analysis

Anticoagulated blood samples were obtained using vacuum tubes with EDTA. PBMCs were prepared from venous blood of BD patients (*n *= 8) and normal controls (*n *= 52) by Ficoll-Hypaque density-gradient centrifugation. Skin was obtained from the skin lesions of patients (*n *= 4) and the scrotum of normal controls (*n *= 22). Total RNA was isolated from skin and PBMCs of the patients or controls by using the QIAamp^® ^RNA Blood Mini kit (QIAGEN Inc., Hilden, Germany) or Qiagen RNeasy Fibrous Tissue Mini Kit (QIAGEN Inc., Hilden, Germany) with treatment of Dnase I, according to the manufacturer's instructions and reversed into cDNA according to the Superscript protocol (SuperScript III First-Strand Synthesize System, Invitrogen, Carlsbad, CA, USA). To compare the mRNA expression of the two protein-coding transcript variants of *UBAC2*, Real-time Quantitative PCR was performed using the Applied Biosystems 7500 System (Applied Biosystems) with the following primers (UBAC2F:5'CCG GCT CCA GTG GGC TCT ACA3'/UBAC2R:5'GGG CGA GCA GGA GGG AGA GG3') for the UBAC2 transcript variant 1 to generate a 81 bp product and (UBAC2F: 5'TAGGAAGTCGTGGCGAGGGAGC3'/UBAC2R: 5'GCCTTGTCTGCTGAC CACCGCT3') for the UBAC2 transcript variant 2 to generate a 84 bp product. The β-actin (β-actinF: 5'GGA TGC AGA AGG AGA TCA CTG3'/*β-actinR:5'CGA TCC ACA CGG AGT ACT TG3'*) was chosen as the internal reference gene to normalize *UBAC2 *expression. RT-PCR conditions were one cycle of 95°C for 10 minutes, followed by 40 cycles in which each cycle included 95°C for 15 sec, 60°C for 1 minute, and 95°C for 15 sec, 60°C for 1 minute, 95°C for 15 sec, 60°C for 15 sec.

### Luciferase Reporter Assay

The whole genome synthesized promoter sequences of *UBAC2 *carrying the T allele or the G allele of SNP rs3825427 were cloned into pGL3-basic vector (Promega, Madison, WI, USA). This vector was then transiently transfected into HEK293 cells. Reporter plasmid was transfected to cells using Lipofectamine reagent (Life Technologies, Grand Island, NY, USA). Transfection efficiency was standardized by cotransfecting with pRL-SV40 (Promega). Luciferase activity was determined after 24 hours incubation using a Luciferase Assay system (Beyotime, Jiangsu, China). For each plasmid construct, three independent transfection experiments were performed, and each was done in triplicate.

### Statistical analysis

Hardy-Weinberg equilibrium (HWE) was tested using the chi-square test and no SNPs showed significant deviation from HWE (*P >*0.05). Odds ratios (OR) and 95% confidence intervals (95% CI) were calculated by using using SPSS version 17.0 (Chicago, IL, USA) to estimate disease risk. Linkage disequilibrium (LD) was examined by using Haploview (version 4.2, Daly Lab at the Broad Institute, Cambridge, MA, USA). To account for multiple testing, the Bonferroni correction was applied. Review Manager 4.2 (Cochrane Collaboration, Oxford, UK) was used to perform meta-analysis. The conditional logistic regression analysis (SAS, 9.13, Cary, NC, USA) was performed to localize the effect and identify the number of independent effects.

## Results

The clinical findings of the BD patients included in our study are shown in Table 2. No statistical difference in the distribution of age and gender was observed between BD patients and controls (*P >*0.05).

### The identification of risk gene for BD by candidate genes association study in Southern China (first-stage study)

Five genes/loci, that is, *LOC100129342*, *KIAA1529*, *CPVL*, *UBASH3B *and *UBAC2*, have been identified as risk factors for BD in Turkish. These five candidate genes/loci were not validated by other ethnic populations. We conducted a candidate gene association study to confirm the association result using 147 BD patients and 951 controls. Our results showed that the rs9513584 polymorphism in*UBAC2 *was associated with the susceptibility to BD (*P*c = 0.018, OR = 1.4, meta-analysis OR = 1.5) (Table 3). There was no association of *LOC100129342*, *KIAA1529, CPVL *and *UBASH3B *with BD in the Chinese cohorts (Table 3).

**Table 3:** The meta- analysis result combined our data and Turkish data

SNP	Nearest gene	Risk allele	Population	N		Risk allele frequency		*P*-value	Pc value	OR (95% CI)
							
				Cases	Controls	Cases	Controls			
rs11206377	*LOC100129342*	G	Turkish^a^	152	170	66.1	51.4	3.0 × 10^-4^	1.5 × 10^-3^	1.8 (1.3 to 2.6)
(1p34)			Chinese	147	951	56.5	53.9	0.410	NS	1.1 (0.9 to 1.4)
			meta-analysis							1.3 (1.1 to 1.6)
rs2061634	*KIAA1529*	G	Turkish	152	170	42.7	26.7	4.2 × 10^-5^	2.1 × 10^-4^	2.0 (1.5 to 2.9)
(9q22)			Chinese	147	951	79.9	80.0	0.971	NS	1.0 (0.7 to 1.4)
			meta-analysis							1.4 (1.1 to 1.7)
rs317711	*CPVL*	C	Turkish	152	170	25.5	13.2	1.0 × 10^-4^	5.0 × 10^-4^	2.2 (1.4 to 3.3)
(7p15-p14)			Chinese	147	951	85.8	84.1	0.739	NS	1.3 (0.9 to 1.9)
			meta-analysis							1.6 (1.2 to 2.1)
rs4936742	*UBASH3B*	T	Turkish	152	170	56.7	43.4	1.5 × 10^-3^	7.5 × 10^-3^	1.7 (1.2 to 2.4)
(11q24)			Chinese	147	951	61.5	63.2	0.590	NS	0.9 (0.7 to 1.2)
			meta-analysis							1.2 (0.9 to 1.4)
rs9513584	*UBAC2*	G	Turkish	152	170	44.4	33.2	5.8 × 10^-3^	0.029	1.6 (1.2 to 2.3)
(13q32)			Chinese	147	951	51.4	42.3	3.6 × 10^-3^	0.018	1.4 (1.1 to 1.8)
			meta-analysis							1.5(1.2 to 1.8)

^a^Data from reference 19; OR (95% CI), Odds ratio (95%CI); NS: not significant; Pc: Bonferroni corrected *P-*value

### Fine-mapping study and validation study of *UBAC2 *association in Chinese cohort

**Figure 1:**
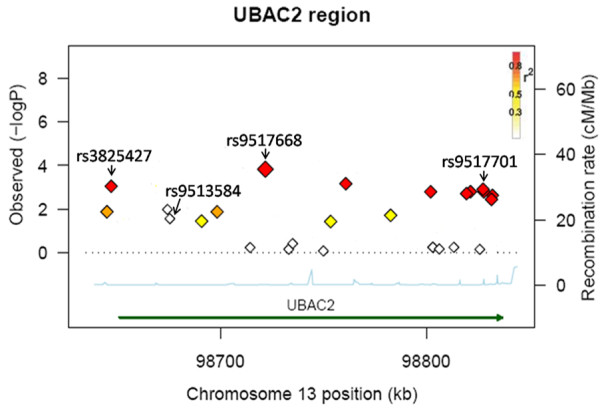
**Genetic association analysis in *UBAC2 *gene region**. Overview of SNPs across the *UBAC2 *gene region in the Chinese cohort. Linkage disequilibrium (*r*^2^) to the most significantly SNP (rs9517668, red diamond) is color-coded (red: *r*^2 ^> 0.8; orange: *r*^2 ^= 0.5-0.8; yellow: *r*^2 ^= 0.2-0.5; white: *r*^2 ^< 0.2). Recombination rates across each region in HapMap CHB are shown in light blue (right *y *axis). The chromosomal locations and relative positions of genes according to hg18 are shown (*x *axis).

The variant in *UBAC2 *showed a consistent association in the Turkish and Chinese cohort, therefore, *UBAC2 *was considered as the most important candidate among the five loci. We, therefore, fine-mapped the *UBAC2 *using 25 SNPs and found that 6 SNPs, that is, rs3825427, rs1927726, rs9517668, rs9554581, rs9517701 and rs9517699, were associated with BD (*P *≤0.05/25 = 0.002) (Table 4, Figure 1 and Additional file 1). We next checked the independence of the evidence for multiple associations within the *UBAC2 *locus by carrying out a conditional logistic regression analysis of the 25 SNPs in *UBAC2*. After control for the genetic effect of rs3825427, none of the 24 SNPs remained significantly associated with Behçet's disease after correction for the number of SNPs tested by the conditional analysis (the smallest *P *= 0.0024 > 0.05/25 = 0.002). Therefore, the multiple associations within the *UBAC2 *locus are not independent.

**Table 4:** Summary of the association of *UBAC2 *SNPs with Behcet's disease in Han Chinese population

		First-stage					Second-stage					Combined study				
				
SNPs	MA	AF		Pc Value	OR	95% CI	AF		Pc	OR	95% CI	AF		Pc	OR	95% CI
													
		Case	Ctrl				Case	Ctrl	Value			Case	Ctrl	Value		
rs3825427	T	116 (39.5)	565 (29.7)	2.2 × 10^-3^	1.5	1.2 to 2.0	248 (37.6)	231 (30.4)	9.3 × 10^-3^	1.4	1.1 to 1.7	364 (38.2)	796 (29.8)	6.9 × 10^-6^	1.5	1.2 to 1.7
rs9517668	T	118 (41.5)	573 (30.1)	1.7 × 10^-3^	1.6	1.2 to 2.0	230 (34.8)	219 (30.9)	0.03	1.3	1.1 to 1.7	348 (36.5)	792 (29.7)	3.3 × 10^-4^	1.4	1.2 to 1.6
rs9517701	G	117 (39.8)	583 (30.7)	5.1 × 10^-3^	1.5	1.2 to 1.9	249 (37.7)	232 (30.3)	9.0 × 10^-3^	1.4	1.1 to 1.7	366 (38.4)	815 (30.5)	2.9 × 10^-5^	1.4	1.2 to 1.7

95% CI, confidence interval; AF, allele frequency; MA, minor allele; OR, odds ratio; Pc: Bonferroni corrected *P*-value.

To further validate the fine-mapping result, we performed a replication study on the selected three SNPs, including rs3825427, rs9517668 and rs9517701, using the West-Southern China cohort. The result showed that all of the three selected SNPs were associated with BD (rs3825427, First-stage *P*c = 2.2 × 10^-3^, OR = 1.5, Second-stage *Pc *= 9.3 × 10^-3^, OR = 1.4, combined *Pc *= 6.9 × 10^-6^, OR = 1.5; rs9517668, First-stage *P*c = 1.7 × 10^-3^, OR = 1.6, Second-stage *Pc *= 0.03, OR = 1.3, combined *Pc *= 3.3 × 10^-4^, OR = 1.4; rs9517701, First-stage *P*c = 5.1 × 10^-3^, OR = 1.5, Second-stage *Pc *= 9.0 × 10^-3^, OR = 1.4, combined *Pc *= 2.9 × 10^-5^, OR = 1.4; respectively) (Table 4).

### Effects of promoter polymorphism of rs3825427 on *UBAC2 *transcriptional level

**Figure 2:**
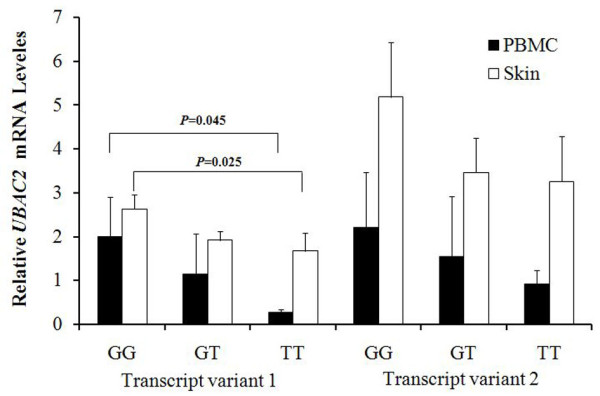
**Comparison of *UBAC2 *variants transcriptional level among different genotypes of rs3825427**. Five GG genotype, four GT genotype, three TT genotype PBMCs samples and four GG genotype, three GT genotype and five TT genotype skin samples were used to examine the expression of *UBAC2 *transcript variant 1 and 2 and each sample were assayed three times. The mean ± SD is given for each genotype from three experiments. Statistical significance was taken when Bonferroni corrected two-tailed *P *< 0.05 using SPSS17.0.

Since the rs3825427 polymorphism is located in the promoter region of *UBAC2 *and bioinformatics analysis revealed that this G/T SNP changed the binding site for transcriptional factors, we performed a series of functional analyses to examine whether rs3825427 polymorphism affected the expression of *UBAC2*. We tested two protein-coding transcript variants of *UBAC2 *in mRNA level with the presence of different genotype (TT/GT/GG) in rs3825427. The mRNA level of *UBAC2 *transcript variant 1 was found decreased in PBMCs and the skin of normal individuals with the TT genotype compared with those with the GG genotype (Bonferroni corrected *P *= 0.045, *P *= 0.025; respectively) (Figure 2). No significant difference was observed in the expression of *UBAC2 *transcript variant 2 between the various genotypes (Figure 2).

**Figure 3:**
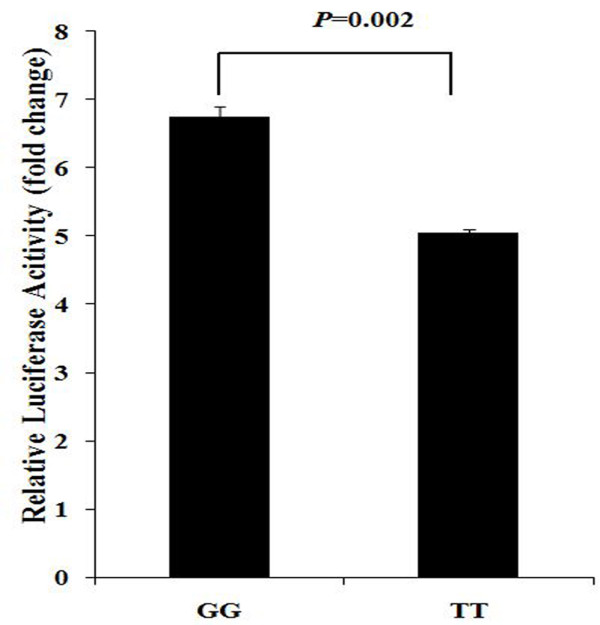
**Effects of the rs3825427 genotype in *UBAC2 *on luciferase activity in cultured HEK 293 cells**. pGL3 luciferase reporter recombinant plasmids containing an *UBAC2 *promoter sequence with the risk allele T or wild-type G allele at SNP rs3825427 were transfected into HEK293 cells. Renilla luciferase plasmid pTK-SV40 was cotransfected with each construct as an internal control for normalization. The mean ± SD is given for each construct from three experiments. Statistical significance was taken when two-tailed *P *< 0.05 using SPSS17.0 independent sample t test.

We subsequently performed a Dual Luciferase Reporter Gene Assay to evaluate whether the promoter sequence carrying different alleles have different promoter activity. The promoter sequences of *UBAC2 *carrying T allele or G allele of SNP rs3825427 were synthesized by Sangon Biotech (Shanghai, P.R. China). Sequencing was performed to validate the result of synthesis and the result showed that only one variant was observed in the location of the rs3825427 polymorphism. The luciferase reporter expression was found to be decreased in the T allele as compared to cells carrying the G allele (*P *= 0.002) (Figure 3).

### Down-regulated expression of *UBAC2 *in PBMCs and skin of BD patients

**Figure 4:**
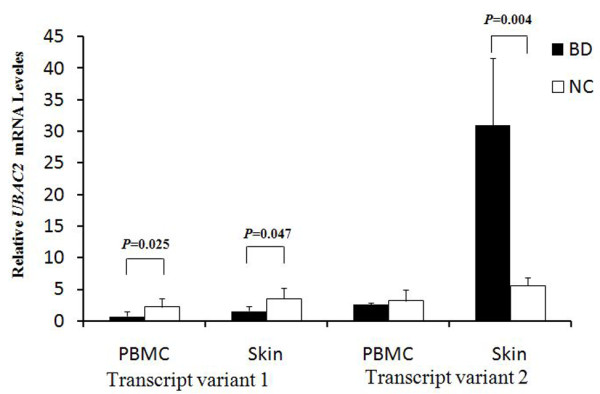
**Real-time RT-PCR analysis of *UBAC2 *transcript variant 1 and 2 mRNA levels between Behçet's patients and normal controls**. *UBAC2 *transcript variant 1 and 2 mRNA levels derived from PBMCs of 23 normal controls and 6 patients or from skin of 9 normal controls and 4 patients. Each RT-PCR was assayed in triplicate. Significance was examined by using SPSS's independent sample t test.

A further study was performed to test whether the mRNA expression of *UBAC2 *was altered in BD. The results showed that the expression of *UBAC2 *was significantly decreased in the PBMCs and skin from BD patients as compared with that observed in normal controls (*P *= 0.025, *P *= 0.047; respectively) (Figure 4). The mRNA expression of UBAC2 transcript variant 2 was significantly increased in skin of BD patients as compared with controls (*P *= 0.004) (Figure 4).

## Discussion

In this study we performed a candidate gene analysis combined with a fine mapping study and the result showed a link of several SNPs in the *UBAC2 *gene with the susceptibility to Behçet's disease in Chinese Han patients. Functional analysis revealed that the risk-associated T allele of rs3825427 significantly down-regulated the expression of *UBAC2 *mRNA. A significantly decreased expression of *UBAC2 *was observed in PBMCs and skin of BD patients compared to normal controls. Our study confirms the association between a single SNP rs9513584 of the *UBAC2 *gene and BD, which was recently reported in a group of Turkish BD patients. Of interest was the observation that the G allele of rs9513584 was associated with the absence of eye disease in the study in Turkish patients. We could not address this observation since all our patients had eye disease.

The *UBAC2 *gene, also known as *PHGDHL1*, is located at 13q32.3. The *UBAC2 *gene encodes an ubiquitination related structural domain. The study reported by Fei *et al. *[19] and their subsequent replication showed the association of *UBAC2 *gene with Behçet's disease and revealed the possible contribution of UBAC2 in the pathogenesis of BD [20]. Our present study successfully replicated the association between UBAC2 and BD in a Chinese cohort. Two other GWAS did not identify the *UBAC2 *gene as the risk gene for Behçet's disease [22,23]. The inconsistent result may be partly explained by the population heterogeneity and the disparate SNP array (Affymetrix SNP array, Santa Clara, CA, USA vs Illumina SNP array, San Diego, CA, USA). In a previous study, we also identified a polymorphism in a ubiquitin-related gene, *SUMO4*, that was associated with the susceptibility to Behçet's disease in a Chinese Han population [13]. Another ubiquitination related gene, *UBE2QL1*, was also found to be associated with BD in a Turkish cohort [23]. Previous studies suggest that ubiquitination reactions are involved in the regulation of receptor tyrosine kinase signaling and may play important roles in the *TNF-α*, *IL-1β*, and TCR-mediated *NF-κB *activation pathway [24,25]. *NF-κB *has been demonstrated to play a crucial role in the pathogenesis of BD through regulating the apoptosis-related factors and increasing the resistance of T cells to apoptosis [26]. All these findings indicate that the ubiquitination-related pathway may have a protective effect in the development of Behçet's disease.

SNP rs3825427 in the *UBAC2 *gene encodes a promoter polymorphism. Our study showed a close association of this SNP with the susceptibility to BD. To determine whether the promoter polymorphism may modulate the expression of this gene, dual luciferase reporter gene assays and real-time PCR analysis were performed and the results showed that the T allele was associated with decreased promoter activity and may down-regulate the expression of *UBAC2*. These findings suggest that the rs3825427 polymorphism may lead to the down-regulation of *UBAC2 *expression. More interestingly, *UBAC2 *transcription was found to be down-regulated in PBMCs and skin of BD patients as compared to controls. Our results are consistent with that observed in mice deficient in the homologous *UBA *domain as reported by Carpino *et al. *[27] They found that *UBA *knockout mice showed increased cytokine production and also exhibited an increased incidence and severity of experimental autoimmune encephalomyelitis (EAE) as compared with wild-type mice [27].

Several possible limitations of the present study merit particular consideration. First, the patients enrolled in our study were recruited from the Eye Department. Because BD usually affects multiple systems, the patients enrolled in this study might, therefore, represent a separate disease population. Second, the patients enrolled in the replication study were recruited only from Chinese Han individuals and the sample size is relatively small. The results presented here need to be confirmed in other ethnic populations and using larger samples. Finally, although molecular biology study provides functional evidence for the rs3825427 polymorphism, this result is generally suggestive and does not exactly explain how the genetic variant translates into physiologic processes and then affects disease susceptibility. Therefore, the association results presented here should be investigated further using more functional experiments.

## Conclusion

In conclusion, our study replicated the association of UBAC2 with BD and identified a promoter SNP of *UBAC2*, rs3825427, to be associated with the increased risk for BD in Chinese. The functional study showed that this SNP may be involved in the development of BD through transcriptional modulation of *UBAC2*.

## Abbreviations

BD: Behçet's disease; EAE: experimental autoimmune encephalomyelitis; EDTA: ethylenediaminetetraacetic acid; GWAS: genome-wide association study; HWE: Hardy-Weiberg equilibrium; LD: Linkage disequilibrium; OR: odds ratio; PBMCs: peripheral blood mononuclear cells; SD: standard deviation; SNP: single-nucleotide polymorphism; SOD: superoxide dismutase; UBAC2: ubiquitin-associated domain containing 2.

## Competing interests

None of the authors has a proprietary or financial interest in any of the products mentioned.

## Authors' contributions

HSP carried out the genotyping, Dual Luciferase Reporter Gene Assay and expression studies and drafted the manuscript. SQM and JZX carried out the genotyping study. CYY, LFZ and CFL participated in the samples collection. HSP and YPZ participated in the design of the study and performed the statistical analysis. HSP and YPZ conceived of the study, and participated in its design and coordination. AK and YPZ revised the manuscript. All authors read and approved the final manuscript.

## Supplementary Material

Additional file 1 **The association of *UBAC2 *gene with Behcet's disease and linkage disequilibrium of *UBAC2 *gene**. Table S1 presented the association analysis of three SNPs in *UBAC2 *gene with Behcet's disease in Han Chinese population. Figure S1 showed linkage disequilibrium plots of *UBAC2 *gene based on the HapMap Phase II dataset for the Han Chinese from the Beijing population by Haploview 4.2 software. Click here for file
