# Supplementary material for: Replication study confirms the association between UBAC2 and Behçet's disease in two independent Chinese sets of patients and controls
Source: Arthritis Res Ther. 2012 Mar 29;14(2):R70. doi: 10.1186/ar3789 (PMC3446441; doi:10.1186/ar3789)
Supplement: Additional file 1 — The association of UBAC2 gene with Behcet's disease and linkage disequilibrium of UBAC2 gene. Table S1 presented the association analysis of three SNPs in UBAC2 gene with Behcet's disease in Han Chinese population. Figure S1 showed linkage disequilibrium plots of UBAC2 gene based on the HapMap Phase II dataset for the Han Chinese from the Beijing population by Haploview 4.2 software. [file ar3789-S1.DOC]

**Table S1 The association analysis of three SNPs in *UBAC2* gene with Behcet’s disease in Han Chinese population**

| SNPs | MA | First-stage | | | | |
| --- | --- | --- | --- | --- | --- | --- |
|  |  | AF | | *Pc* Value | OR | 95%CI |
|  |  | Case (147) | Control (951) |  |  |  |
| rs9554581 | T | 118(40.1) | 591(31.1) | 0.0057 | 1.5 | 1.2-1.9 |
| rs9517699 | T | 118(40.1) | 571(30.0) | 0.0015 | 1.6 | 1.2-2.0 |
| rs1927726 | G | 114(38.8) | 565(29.7) | 0.0054 | 1.5 | 1.2-1.9 |

Pc: Bonferroni corrected P value

**Figure S1 The linkage disequilibrium plots of *UBAC2* gene based on the HapMap Phase II dataset for the Han Chinese from Beijing population by Haploview 4.2 software**

**
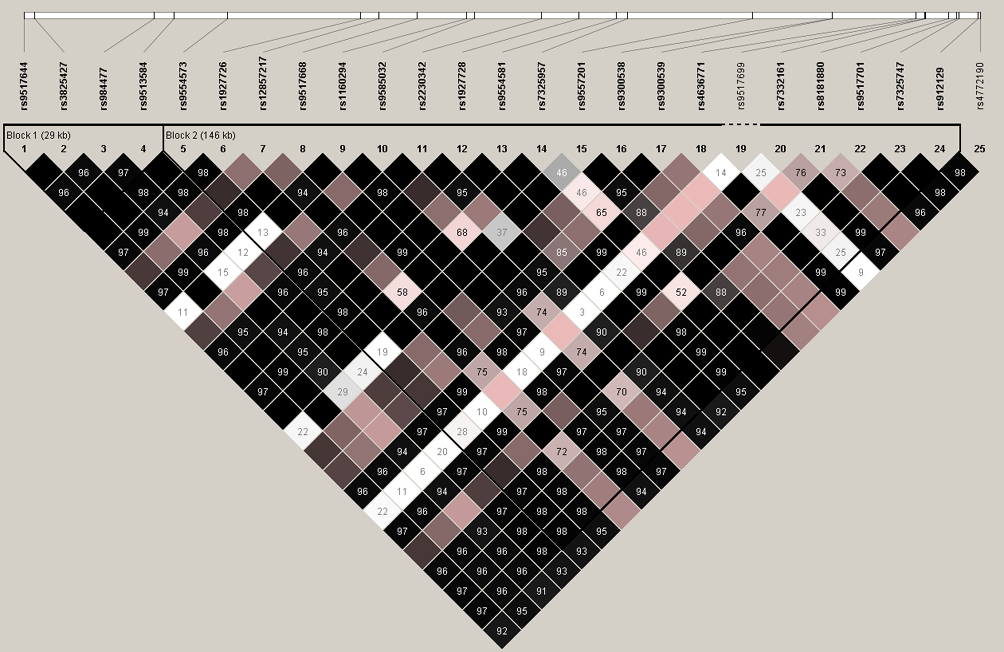
**
